# Association of alpha1a-adrenergic receptor polymorphism and blood pressure phenotypes in the Brazilian population

**DOI:** 10.1186/1471-2261-8-40

**Published:** 2008-12-23

**Authors:** Silvia R Freitas, Alexandre C Pereira, Marcilene S Floriano, José G Mill, José E Krieger

**Affiliations:** 1 Laboratory of Genetics and Molecular Cardiology, Heart Institute/InCor, University of São Paulo Medical School, São Paulo, Brazil; 2 Laboratory of Human Genetics, Oswaldo Cruz Institute/FIOCRUZ, Rio de Janeiro, Brazil; 3 Department of Physiology, Espírito Santo Federal University, Vitória, Brazil

## Abstract

**Background:**

The alpha1A-adrenergic receptor (α_1A_-AR) regulates the cardiac and peripheral vascular system through sympathetic activation. Due to its important role in the regulation of vascular tone and blood pressure, we aimed to investigate the association between the Arg347Cys polymorphism in the α_1A_-AR gene and blood pressure phenotypes, in a large sample of Brazilians from an urban population.

**Methods:**

A total of 1568 individuals were randomly selected from the general population of the Vitória City metropolitan area. Genetic analysis of the Arg347Cys polymorphism was conducted by polymerase chain reaction/restriction fragment length polymorphism. We have compared cardiovascular risk variables and genotypes using ANOVA, and Chi-square test for univariate comparisons and logistic regression for multivariate comparisons.

**Results:**

Association analysis indicated a significant difference between genotype groups with respect to diastolic blood pressure (p = 0.04), but not systolic blood pressure (p = 0.12). In addition, presence of the Cys/Cys genotype was marginally associated with hypertension in our population (p = 0.06). Significant interaction effects were observed between the studied genetic variant, age and physical activity. Presence of the Cys/Cys genotype was associated with hypertension only in individuals with regular physical activity (odds ratio = 1.86; p = 0.03) or younger than 45 years (odds ratio = 1.27; p = 0.04).

**Conclusion:**

Physical activity and age may potentially play a role by disclosing the effects of the Cys allele on blood pressure. According to our data it is possible that the Arg347Cys polymorphism can be used as a biomarker to disease risk in a selected group of individuals.

## Background

Increased blood pressure constitutes a major risk factor for cardiovascular diseases, including kidney and cerebrovascular diseases[1,2]. Although the underlying molecular mechanisms remain largely elusive, it is well known that blood pressure is tightly regulated through a net of complex interrelationships between several physiological systems[3]. In addition, several well-designed studies have provided strong evidence that blood pressure variability is largely determined by genetic factors[4,5].

The alpha1A-adrenergic receptor (α_1A_-AR) regulates the cardiac and peripheral vascular system through sympathetic activation. Because of this characteristic, the α_1A_-AR is considered an important participant in blood pressure homeostasis[6]. The human α_1A_-AR gene (*ADRA1A*), located on chromosome 8q21[7], have an Arg347Cys polymorphism (rs1048101, previously described as Arg492Cys) located in the region coding for the carboxyl terminus of the receptor[8]. The arginine (Arg) → cysteine (Cys) substitution at the amino acid position 347 can confer a palmitoylation site and may modulate the cellular localization of the protein[8]. This variant has no apparent effect on the functional properties *in vitro*[8], and an initial report found no association between this polymorphism and hypertension[9]. However, recent studies showed that the Cys allele was associated with relatively lower hypertension prevalence in a Chinese population[10], and its carriers had a significantly greater blood pressure decrease with short-term Ibersatan treatment in a sample of Chinese hypertensive individuals[11]. These results suggested that genetic variations in *ADRA1A *could modulate cardiac or vascular sympathetic tone and might contribute to the pathogenesis of hypertension and cardiovascular disease.

In this report, we aimed to investigate the association between the Arg347Cys polymorphism, environmental risk factors and blood pressure phenotypes, in a large sample of Brazilians randomly selected from an admixed urban population.

## Methods

### Study Population

A cross-sectional study of risk factors for cardiovascular diseases was performed in the urban population of Vitória, Brazil, using the WHO-MONICA project guidelines[12]. A sample of 2044 individuals (from an eligible population of 137330) of either gender, 25 to 64 years of age, was chosen according to the nearest birthday after a random selection of domiciles. The recruitment process occurred from April, 1999 to November, 2000.

Participants (n = 1573) attended the clinic visit and the physical examination emphasized measurement of height, weight, and blood pressure. Major cardiovascular risk factors, such as, obesity phenotypes, ethnicity, smoking status, amount of physical activity, hypertension and metabolic syndrome, were also evaluated. Laboratorial analyses were conduced to evaluate blood glucose, total-cholesterol, lipoprotein fraction, and triglycerides.

This study was approved by Ethics Committee for Research on Human Subject of the Universidade Federal do Espírito Santo, and all subjects gave written informed consent to participate.

### Anthropologic Investigation and Biochemical Measurements

Presence of traditional cardiovascular risk factors was determined using the criteria standardized by The Seventh Report of the Joint National Committee on Prevention, Detection, Evaluation, and Treatment of High Blood Pressure[13]. Weight and height were measured according to a standard protocol, with participants wearing light clothing and no shoes. Height was measured in centimeters and weight in kilograms using a calibrated balance. Body mass index (BMI) (weight in Kg/height in meters^2^) was calculated and overweight or obesity defined as BMI ≥ 25 or 30 Kg/m^2^, respectively. All participants were also submitted to a racial classification according to a validated questionnaire for the Brazilian population[14,15]. Subjects were classified as Caucasian or African-descent according to a set of phenotypic characteristics (skin color, hair texture, shape of the nose, aspect of the lip, and jaw position). On the basis of these characteristics, mulattos are considered racially mixed subjects. Individuals who had ever smoked more than five cigarettes per day for the least a year were classified as smokers, and sedentary lifestyle was defined as exercise less than one hour, three times a week.

According to the Third Report of the National Cholesterol Education Program criteria[16], metabolic syndrome was defined when any two or more of following risk determinant were present: (1) abdominal obesity (waist circumference ≥ 102 cm in men, and ≥ 88 cm in women); (2) fasting glucose ≥ 100 mg/dL, (3) hypertension (systolic blood pressure ≥ 130 mmHg and/or diastolic blood pressure ≥ 85 mmHg in at least two measurement or the current use of anti-hypertensive medication) and (4) total-cholesterol ≥ 200 mg/dL, triglycerides ≥ 150 mg/dL, LDL-cholesterol ≥ 130 mg/dL and HDL-cholesterol ≤ 40 mg/dL. Blood glucose, total cholesterol, lipoprotein fractions, and triglycerides were assayed by standard techniques in 12-hour fasting blood samples.

### Determination of blood pressure phenotypes

Blood pressure was measured using a standard mercury sphygmomanometer on the left arm after 5 minutes rest, in the sitting position. Systolic and diastolic blood pressures were calculated from three readings, with a minimal interval of 5 minutes and the average value was used in the analysis. Hypertension was defined as mean systolic blood pressure (SBP) of ≥ 130 mmHg and/or diastolic blood pressure (DBP) of ≥ 85 mmHg or the current use of anti-hypertensive medication[16]. This definition was used to standardize the hypertension classification in both hypertensive individual and metabolic syndrome subjects.

### Genotyping Protocols

Genomic DNA was extracted from leukocytes in samples of whole blood, following a standard salting-out technique[17]. Genotypes were detected by polymerase chain reaction (PCR) followed by restriction fragment length polymorphism (RFLP) analysis as previously described[18]. Quality control for these assays was assessed by randomly selecting 60 samples to be re-genotyped by two independent technicians.

### Statistical Analysis

Comparisons of continuous variables were performed by Student unpaired *t*-test, while χ^2 ^tests were used for categorical variables. Allele and genotype frequencies among the study participants were calculated using the StatView for Windows statistical program (version 5.0). The goodness of fit for normal distribution was evaluated using the Kolmogorov-Smirnov test. Hardy-Weinberg equilibrium for the distribution of the genotypes was estimated by the χ^2 ^test using the StatView program. The effects of ethnicity on blood pressure status and genotype frequencies were analyzed by χ^2 ^tests.

Unpaired *t *test and ANOVA were used to investigate the association between genotype and the different phenotypes studied. The association between Arg347Cys polymorphism and blood pressure phenotypes was assessed by analysis of odds ratio (OR), respective 95% confidence intervals (CI) and two-tailed p value (Epi-Info statistical program, version 3.2.2). Genetic models of action of the studied variants were constructed by combining genotypes (i.e., recessive model = homozygous for the Cys allele).

Association between a particularly chosen genetic model, common risk factors (gender, age, obesity, ethnicity, smoking status, sedentary lifestyle, total-cholesterol, lipoprotein fractions, triglycerides, blood glucose, metabolic syndrome) and blood pressure phenotype was examined with simple and multiple logistic regressions. These analyses were conduced using the SPSS program (version 12.0).

Quantitative variables were expressed as the mean ± standard deviation, and *p *values < 0.05 on a two-tailed test were considered statistically significant.

## Results

### Demographic and Genetic Structure Data

Table 1 summarizes the demographic data stratified per Arg347Cys genotypes. In all ethnic groups, allele and genotype frequencies were in accordance with the Hardy-Weinberg equilibrium (*p *> 0.05). Genotypes were differently distributed regarding ethnicity (*p *< 0.001). This difference was due to a higher frequency of the Cys allele in African-descendent individuals as compared to Caucasian-descendent ones.

**Table 1:** Baseline characteristics and clinical data for conventional risk factors per genotype.

Parameters		Arg/Arg	Arg/Cys	Cys/Cys	p
N° individuals		287	813	473	
Anthropologic characteristics					
Gender, Male, %		44.8	43.7	46.8	0.262
Age, years		45.0 ± 11.5	44.9 ± 10.6	44.4 ± 10.8	0.721
BMI, kg/m^2^		26.4 ± 5.0	26.1 ± 5.0	26.5 ± 4.6	0.327
Waist circunference, cm	Male	90.5 ± 12.6	88.5 ± 11.2	89.7 ± 10.7	0.212
	Female	83.3 ± 12.8	83.6 ± 12.5	84.9 ± 13.2	0.257
Obesity, %		55.1	53.4	60.3	0.058
Ethnicity, %	Caucasian	44.0	35.0	31.0	
	Mulatto	48.1	55.0	56.2	0.000
	Negroid	7.9	10.0	12.8	
Smoking, %		23.7	22.5	20.3	0.737
Sedentary Lifestyle, %		70.8	76.0	73.5	0.221
Blood pressure					
SBP, mmHg		125.8 ± 20.4	127.9 ± 22.0	129.2 ± 22.5	0.122
DBP, mmHg		82.6 ± 13.2	84.1 ± 14.1	85.2 ± 14.2	0.044
Pulse Pressure, mmHg		43.2 ± 14.0	43.8 ± 13.3	43.9 ± 14.3	0.748
Hypertension, %		40.1	41.6	45.5	0.263
Blood biochemistry					
Total Cholesterol, mg/dL		215.7 ± 45.9	214.5 ± 45.0	213.6 ± 53.4	0.838
LDL-Cholesterol, mg/dL		142.9 ± 39.9	142.5 ± 39.0	141.4 ± 41.1	0.839
HDL-Cholesterol, mg/dL		44.7 ± 13.0	46.0 ± 12.6	44.7 ± 11.5	0.109
Triglicerides, mg/dL		150.2 ± 162.6	133.0 ± 102.0	138.2 ± 143.2	0.149
Glucose, mg/dL		105.8 ± 31.1	104.6 ± 32.0	105.1 ± 32.8	0.867
Glucose intolerance, %		20.2	21.4	22.9	0.851
Metabolic syndrome, %		28.9	23.3	27.0	0.112

### Association Between Blood Pressure Phenotypes and *ADRA1A *Genotypes

Association analysis indicated a significant difference between genotype groups with respect to diastolic blood pressure and a tendency towards increased systolic blood pressure associated with the presence of the Cys allele using an additive mode of action (Table 1).

Considering a recessive mode of action for the Cys allele (Cys/Cys vs. Arg/Arg + Arg/Cys individuals), presence of the Cys/Cys genotype was marginally associated with hypertension in our population (OR = 1.334; CI = 0.995–1.532; *p *= 0.056). After adjustment for gender, age, and ethnicity, presence of the risk allele in homozygosis was not significant (OR = 1.21; CI = 0.96–1.53; *p *= 0.11).

**Figure 1:**
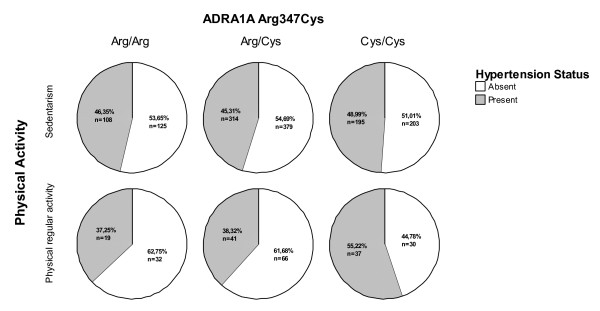
**Hypertension prevalence according to physical activity and Arg347Cys genotypes**.

Exploring potential interaction effects between the Arg347Cys polymorphism and other determinants of blood pressure, we have interestingly observed a context-dependent effect of the polymorphism regarding physical activity (Figure 1). In this scenario, the Arg347Cys polymorphism is associated with hypertension only in individuals with regular physical activity. In this sub-population of fitted individuals, we observed that the hypertensive status was associated with Cys/Cys genotype (OR = 1.86, CI = 1.06–3.26, *p *= 0.03; adjusted for gender, obesity, ethnicity, smoking status, dyslipidemia, diabetes and metabolic syndrome gender). We did not find evidence for an interaction effect between the studied genetic variant and obesity (*p *= 0.23), smoking status (*p *= 0.66), total-cholesterol (*p *= 0.46), LDL-cholesterol (*p *= 0.74), HDL-cholesterol (*p *= 0.71), triglycerides (*p *= 0.84), glucose intolerance (*p *= 0.84) or metabolic syndrome (*p *= 0.67).

**Figure 2:**
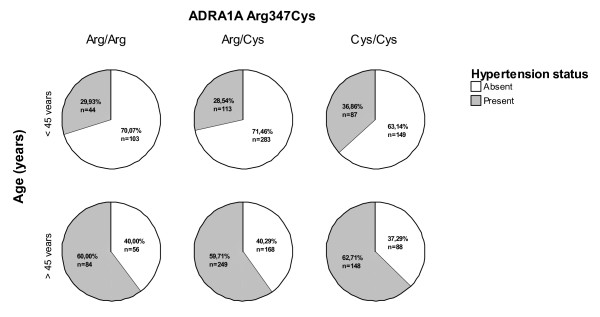
**Hypertension prevalence according to age and Arg347Cys genotypes**.

Another way of testing this potentially relevant interaction effect is to study the differential effect on hypertensive status modulated by the Arg347Cys variant comparing populations with different ages. Here, we have hypothesized that individuals of younger age (and conceivably fitter) may show a different effect of the Cys/Cys genotype when compared to the same genotype effect in individuals from a higher age group. In Figure 2, we show the different prevalence of hypertension according with the Arg347Cys genotypes in different sub-groups defined by the median age of our population, 45 years. Interestingly, in individuals with less than 45 years there is a clear association involving the Cys/Cys genotype hypertension phenotype (OR = 1.27; CI = 1.01–1.59; *p *= 0.04). On the contrary, despite a higher prevalence of hypertension in the older individuals sample, there is no clear association between the Cys/Cys genotype and hypertension (*p *= 0.46).

## Discussion

The data presented in this study provide evidence for a discrete association between Arg347Cys polymorphism and blood pressure related phenotypes in the general population studied. Nevertheless, a stronger association between the Cys allele and hypertension was clearly observed in the subpopulations of fitted and young individuals. These findings suggest that the effect of this allele in blood pressure regulation is only evident when taking into account physical fitness. Physical activity, potentially, may play a role by disclosing the effects of the Cys allele on blood pressure, consequently uncovering the association between them.

Alpha1-adrenergic receptors (α_1A_-AR, α_1B_-AR e α_1C_-AR) are G protein-coupled transmembrane receptors that mediate actions in the sympathetic nervous system through the binding of catecholamines[19]. Among three subtypes of human α_1_-ARs, the α_1A_-AR is the predominant subtype in vascular smooth muscle. Genetically engineered knock-out mice for the α_1A_-AR have demonstrated that this subtype has a vasopressor role in resistance arteries and is required to maintain normal arterial blood pressure[20]. Although functional differences between the Arg347 and Cys347 receptors have yet to be identified, the potential relevance of α_1A_-AR genetic variation to multiple pathophysiological conditions have been examined with negative results. However, most studies were carried in small sample sizes and underpowered to explore small effect-sizes and gene-environment interactions[8,9,11,18,21].

Not in contrast with these findings, a recent study by Jiang and co-authors [11] showed that α_1A_-AR Cys allele carriers had a significantly greater blood pressure response to short-term Irbesartan treatment. Snapir and co-authors [22] observed that young healthy subjects homozygous for the Cys allele had a tendency to increased systolic blood pressure and longer electrocardiogram RP interval before and during the adrenaline infusion. Moreover, Iacovello and co-workers [23] described a strong association between the Cys allele and autonomic control of heart rate in healthy individuals. The observed association between the Cys allele and hypertension in a subpopulation of young individuals or with regular physical activity in the present study may be in part responsible for the physiological implications of this polymorphism. Certainly, in the current research design, we could not exclude the possibility that the Arg347Cys polymorphism or other functional genetic variant in linkage disequilibrium with it may have a potential clinical implication for α_1A_-AR-mediated physiology in relation to blood pressure modulation. In this case, haplotype analysis may provide further information on this issue.

The alpha1A-adrenergic receptor mediates vasoconstriction and plays an important role in the regulation of vascular tone. It is well documented that sympathetic nerve stimulation produces substantial vasoconstriction in skeletal muscle via α1- and α2-adrenergic receptors[24]. Similarly, both receptors contribute to sympathetic vasoconstriction in skeletal muscle at rest and during exercise[25]. In addition, the mechanisms mediating post-exercise hypotension may indeed require the participation of the alpha-adrenergic receptor system[26].

Although the study of physiological control systems known to contribute to the regulation of blood pressure during exercise had shed light on the complex interrelations between hypertension and physical activity, the molecular basis of the relationship between these two conditions remain poorly understood. It is interesting to note that we were able to observe an association between the Cys/Cys genotype and hypertension only in individuals with regular physical activity. This fact may relates to the important role of alpha-adrenergic receptors in modulating blood pressure homeostasis during and after exercise[25].

Our study has potential limitations: (1) perhaps a case-control study design may be more informative to evaluate the hypertension susceptibility than the present cross-sectional study; (2) blood pressure measurements were taken in only one visit instead of the more accepted procedure of three different medical visits; (3) our analysis has shown the existence of significant population structure and the statistical power associated to stratified ethnic subgroup analysis was significantly reduced in comparison with that associated to the whole population analysis; (4) we used the III Report of the National Cholesterol Education Program criteria to classify hypertension status. This procedure was applied in order to standardize the hypertension classification in both hypertensive individual and metabolic syndrome subjects. In this context, the present observations should be considered cautiously in relation to the pathology of hypertension and cardiovascular disease; (5) the functional role of Arg347Cys polymorphism is still unknown, but epidemiological studies have been suggesting that this molecular variant may be associated with blood pressure controlling mechanisms and hypertension status; (6) we have only genotyped the Arg347Cys SNP in the *ADRA1A *(the most common non-synonymous naturally occurring SNP in the α_1A_-AR) and other(s) SNP(s) may, in fact, be more relevant for the reported association[19]. Nevertheless, at least for the studied population, we were able to show that the studied genetic marker has predictive power to stratify individuals according to hypertension risk. It remains to be tested whether different markers in the same gene will also be predictive of hypertension risk.

## Conclusion

Present data suggested that, although *ADRA1A *Cys allele may have a discrete effect in individuals from the general population, it might become a relevant marker of the hypertension risk in individuals younger than 45-years. Moreover, these findings provide further insights for the understanding of the complex modulation of blood pressure by exercise activity. Future work should be carried-out in order to understand the context-dependent effect of the Arg347Cys variant regarding physical activity and to shed light on the potential role of *ADRA1A *genotype for predicting exercise-induced hypotension.

## Competing interests

The authors declare that they have no competing interests.

## Authors' contributions

ACP conceived of the study, and participated in its design and coordination and in the statistical analysis and to draft the manuscript. SRF participated in the statistical analysis and helped to draft the manuscript. MSF carried out the molecular genetic analysis. JGM participated in the design of the study and performed the clinical analysis. JEK participated in the design of the study and coordination. All authors read and approved the final manuscript.

## Pre-publication history

The pre-publication history for this paper can be accessed here:



## References

[B1] He J, Whelton PK (1999). Elevated systolic blood pressure and risk of cardiovascular and renal disease: overview of evidence from observational epidemiologic studies and randomized controlled trials. Am Heart J.

[B2] Kannel WB (2000). Elevated systolic blood pressure as a cardiovascular risk factor. Am J Cardiol.

[B3] Lifton RP, Gharavi AG, Geller DS (2001). Molecular mechanisms of human hypertension. Cell.

[B4] Gu C, Borecki I, Gagnon J, Bouchard C, Leon AS, Skinner JS, Wilmore JH, Rao DC (1998). Familial resemblance for resting blood pressure with particular reference to racial differences: preliminary analyses from the HERITAGE Family Study. Hum Biol.

[B5] Rotimi CN, Cooper RS, Cao G, Ogunbiyi O, Ladipo M, Owoaje E, Ward R (1999). Maximum-likelihood generalized heritability estimate for blood pressure in Nigerian families. Hypertension.

[B6] Rudner XL, Berkowitz DE, Booth JV, Funk BL, Cozart KL, D'Amico EB, El-Moalem H, Page SO, Richardson CD, Winters B (1999). Subtype specific regulation of human vascular alpha(1)-adrenergic receptors by vessel bed and age. Circulation.

[B7] Hoehe MRBWH, Schwinn DA, Hsieh W-T (1992). A two-allele PstI RFLP for the alpha-1C adrenergic receptor gene (ADRA1C). Hum Mol Genet.

[B8] Shibata K, Hirasawa A, Moriyama N, Kawabe K, Ogawa S, Tsujimoto G (1996). Alpha 1a-adrenoceptor polymorphism: pharmacological characterization and association with benign prostatic hypertrophy. Br J Pharmacol.

[B9] Xie HG, Kim RB, Stein CM, Gainer JV, Brown NJ, Wood AJ (1999). Alpha1A-adrenergic receptor polymorphism: association with ethnicity but not essential hypertension. Pharmacogenetics.

[B10] Gu DGD, Snieder H, He J, Chen S, Huang J, Li B, Chen R, Quiang B (2006). Association of alpha1A adrenergic receptor gene variants on chromosome 8q21 with human stage 2 hypertension. J Hypertension.

[B11] Jiang S, Mao G, Zhang S, Hong X, Tang G, Li Z, Liu X, Zhang Y, Wang B, Xu X (2005). Individual and joint association of alpha1A-adrenergic receptor Arg347Cys polymorphism and plasma irbesartan concentration with blood pressure therapeutic response in Chinese hypertensive subjects. Clin Pharmacol Ther.

[B12] (1988). The World Health Organization MONICA Project (monitoring trends and determinants in cardiovascular disease): a major international collaboration. WHO MONICA Project Principal Investigators. J Clin Epidemiol.

[B13] Chobanian AV, Bakris GL, Black HR, Cushman WC, Green LA, Izzo JL, Jones DW, Materson BJ, Oparil S, Wright JT (2003). The Seventh Report of the Joint National Committee on Prevention, Detection, Evaluation, and Treatment of High Blood Pressure: the JNC 7 report[see comment][erratum appears in JAMA. 2003 Jul 9;290(2):197]. JAMA.

[B14] Lessa I, Fonseca J (1997). Raca, aderencia ao tratamento e/ou consultas e controle da hipertensao arterial. Arquivos Brasileiros de Cardiologia.

[B15] McKenzie K, Crowcroft NS (1996). Describing race, ethnicity, and culture in medical research[see comment][comment]. BMJ.

[B16] (2001). Executive Summary of The Third Report of The National Cholesterol Education Program (NCEP) Expert Panel on Detection, Evaluation, And Treatment of High Blood Cholesterol In Adults (Adult Treatment Panel III). JAMA.

[B17] Miller SA, Dykes DD, Polesky HF (1988). A simple salting out procedure for extracting DNA from human nucleated cells. Nucleic Acids Research.

[B18] Mochtar CA, Laan W, Van Houwelingen KP, Franke B, De La Rosette JJ, Schalken JA, Kiemeney LA (2006). Polymorphisms in the alpha1A-adrenoceptor gene do not modify the short- and long-term efficacy of alpha1-adrenoceptor antagonists in the treatment of benign prostatic hyperplasia. BJU Int.

[B19] Lei B, Morris DP, Smith MP, Svetkey LP, Newman MF, Rotter JI, Buchanan TA, Beckstrom-Sternberg SM, Green ED, Schwinn DA (2005). Novel human alpha1a-adrenoceptor single nucleotide polymorphisms alter receptor pharmacology and biological function. Naunyn Schmiedebergs Arch Pharmacol.

[B20] Rokosh DG, Simpson PC (2002). Knockout of the alpha 1A/C-adrenergic receptor subtype: the alpha 1A/C is expressed in resistance arteries and is required to maintain arterial blood pressure. Proc Natl Acad Sci USA.

[B21] Hsu JW, Wang YC, Lin CC, Bai YM, Chen JY, Chiu HJ, Tsai SJ, Hong CJ (2000). No evidence for association of alpha 1a adrenoceptor gene polymorphism and clozapine-induced urinary incontinence. Neuropsychobiology.

[B22] Snapir A, Koskenvuo J, Toikka J, Orho-Melander M, Hinkka S, Saraste M, Hartiala J, Scheinin M (2003). Effects of common polymorphisms in the alpha1A-, alpha2B-, beta1- and beta2-adrenoreceptors on haemodynamic responses to adrenaline. Clinical Science.

[B23] Iacoviello M, Forleo C, Sorrentino S, Romito R, De Tommasi E, Lucarelli K, Guida P, Pitzalis MV (2006). Alpha- and beta-adrenergic receptor polymorphisms in hypertensive and normotensive offspring. Journal of Cardiovascular Medicine.

[B24] Ohyanagi M, Faber JE, Nishigaki K (1991). Differential activation of alpha 1- and alpha 2-adrenoceptors on microvascular smooth muscle during sympathetic nerve stimulation. Circ Res.

[B25] Buckwalter JB, Clifford PS (1999). alpha-adrenergic vasoconstriction in active skeletal muscles during dynamic exercise. Am J Physiol.

[B26] Rao SP, Collins HL, DiCarlo SE (2002). Postexercise alpha-adrenergic receptor hyporesponsiveness in hypertensive rats is due to nitric oxide. Am J Physiol Regul Integr Comp Physiol.

